# HT-Net: A Task-Specific Dual-Branch Network for Tumor Segmentation in Histopathological Images

**DOI:** 10.3390/biomimetics11100739

**Published:** 2026-10-14

**Authors:** Lijuan Ren, Hang Chen, Jun Yang, Lei He, Dan Tang

**Affiliations:** 1 School of Software Engineering, Chengdu University of Information Technology, Chengdu 610225, China3240707065@stu.cuit.edu.cn (H.C.);; 2 Sichuan Provincial Big Data Technology Services Center, Chengdu 610020, China

**Keywords:** tumor segmentation, histopathological imaging, dual-branch encoder, multi-scale attention, task-specific supervision

## Abstract

Breast cancer, the most prevalent malignancy among women globally, requires precise assessment of lymph node metastases in whole-slide images (WSIs) for accurate clinical staging and therapeutic planning. However, manual assessment is laborious and prone to inter-observer variability. Inspired by biomimetic principles of functional specialization and complementary visual perception, we propose Head–Tail Network (HT-Net), a novel dual-branch encoder network for histopathological image segmentation. HT-Net employs a HeadBranch with Squeeze-and-Excitation (SE) attention to prioritize background feature selection and a TailBranch with a novel Hybrid Multi-Scale Attention (HMSA) module to resolve complex tumor boundaries. Features from both branches are integrated through a spatially adaptive fusion module and reconstructed using a U-Net-style decoder. A task-specific supervision loss further enforces functional specialization between branches and mitigates class imbalance. Experiments demonstrate that HT-Net outperforms U-Net, UNet++, UCTransNet, VM-UNet, and nnWNet. On Camelyon16, HT-Net achieved 89.37% mDice and 82.05% mIoU, surpassing nnWNet by 0.86 and 1.08 percentage points, respectively. On Glas, it achieved 93.04% mDice and 86.95% mIoU, exceeding nnWNet by 0.30 and 0.47 percentage points. These results demonstrate the effectiveness of the biomimetic-inspired functional specialization strategy for automated histopathological image segmentation.

## 1. Introduction

Breast cancer remains the most prevalent malignant neoplasm among women worldwide. Global cancer statistics indicate that in 2020, there were approximately 2.26 million new cases, surpassing lung cancer as the leading cause of cancer-related mortality among females [1,2]. Because lymph node metastasis significantly dictates clinical staging and therapeutic strategy, the precise evaluation of sentinel lymph nodes (SLNs) has become a cornerstone of pathological diagnosis [3]. Currently, pathological analysis utilizing whole-slide imaging (WSI) is the gold standard for assessing SLN status [4]. However, this approach relies heavily on the manual, region-specific examination by specialized pathologists. A single WSI can contain hundreds of thousands of pixels, and a comprehensive inspection is labor-intensive and time-consuming. Furthermore, diagnostic consistency is often compromised by a pathologist’s subjective experience and fatigue, leading to a non-negligible risk of misdiagnosis or overlooked metastases [5]. Meanwhile, these efficiency bottlenecks are exacerbated by a global shortage of pathologists. Moreover, the acquisition of high-quality, expert-annotated datasets is prohibitively expensive [6], creating a data bottleneck for deep learning applications. Consequently, automated pathological image analysis enabled by deep learning has gradually emerged as a research frontier [7], offering a promising pathway to enhance diagnostic efficiency and mitigate subjective bias. These challenges highlight the need for automated analysis methods capable of efficiently processing complex pathological visual information while preserving clinically meaningful morphological characteristics. In this context, biomimetics provides a useful conceptual framework for developing intelligent visual analysis systems. Biological perception commonly relies on hierarchical processing, selective attention, and functional specialization to extract complementary information from complex environments. Translating these principles into computational architectures offers a promising strategy for handling the heterogeneous and highly structured visual information encountered in histopathological images.

Early investigations into automated analysis primarily adopted the patch-level classification paradigm, where WSIs are segmented into fixed-size local regions for individual assessment. For instance, Wang D et al. [3] employed this approach to achieve pixel-level detection of lymph node metastasis. Subsequently, end-to-end methodologies utilizing Multiple Instance Learning (MIL) sought to capture global context; Wang Z et al. [8] introduced a Transformer-guided MIL framework that characterizes contextual relationships among patches via a self-attention mechanism and leverages transfer learning to its predictive accuracy, even with scarce labeled data. Nonetheless, the paradigms—whether patch-based or MIL-based—fundamentally generate “region-level” or “slice-level” probabilities. Such outputs fail to precisely represent tumor morphological contours and invasion boundaries [9]. From a biomimetic perspective, this limitation can be viewed as a consequence of relying primarily on a single level of visual representation, whereas biological perception achieves robust recognition through complementary processing of global context and fine-grained local information. This technique in SLN diagnosis, clinicians require more than a binary “presence or absence” result, they must evaluate tumor morphology, distribution, and extent—features that are difficult to capture with weakly supervised MIL or patch-level classification. Therefore, moving toward pixel-level segmentation not only provides finer pathological characterization but also creates an opportunity to incorporate biomimetic principles of complementary feature extraction and adaptive information integration into network design. As a result, research has shifted toward the more complex task of tumor segmentation.

Tumor segmentation in histopathological images aims to assign a categorical label (e.g., tumor, stroma, lymphocytes) to each pixel, providing clinicians with a comprehensive “tumor distribution heatmap.” This technique enhances analytical granularity from the “block level” to the “pixel level”, enabling a more precise delineation of tumor extent. The encoder–decoder architecture has become the predominant framework for this task, owing to its comprehensive pixel prediction functionality. Specifically, U-Net [10]—with its symmetric architecture and skip connections—effectively addresses the challenge of limited training data in medical imaging. Subsequent advancements have expanded this framework: UNet++ introduced nested dense skip connections to bridge the semantic gap [11], Attention U-Net utilized gated attention to highlight fine-scale features [12], and TransUNet [13] and Swin-UNet [14] integrated Transformers to enhance global modeling. While attention modules like SE-Net [15] and CBAM [16] have further improved feature discriminability in both channel and spatial dimensions, a core contradiction remains unresolved in current methodologies.

Existing methods typically rely on the implicit assumption that features across different image regions can be uniformly extracted through a single encoding strategy. In pathological tumor segmentation, this assumption is often invalid due to three primary challenges: (1) tumor regions and adjacent normal tissue exhibit vast differences in texture, color, and morphology, which a single-branch encoder struggles to capture simultaneously. (2) tumor regions often occupy only a small fraction of the slide area, causing the supervisory signal to be dominated by the background and constraining the model’s perception of tumorous areas. (3) the transition between tumors and normal tissue is often characterized by gradual and blurred edges, which are difficult for static receptive fields to resolve.

To address these challenges, we introduce Head–Tail Network (HT-Net), a dual-branch encoder network that optimizes tumor segmentation through innovations in architectural design, feature modeling, and supervision. The primary contributions of this study are as follows:

1. Dual-Branch Parallel Encoder: We propose an architecture comprising a HeadBranch (background extraction) and a TailBranch (tumor extraction). These branches utilize distinct attention mechanisms tailored to their respective regions, replacing conventional homogeneous extraction to address regional feature heterogeneity.
2. Hybrid Multi-Scale Attention (HMSA): We developed an HMSA module that integrates parallel channel attention pathways with varying compression ratios and spatial attention branches using dilated convolutions. This allows for the adaptive representation of channel dependencies and spatial context across multiple scales, significantly enhancing boundary detection.
3. Task-Specific Supervision: We implement a specialized loss function where the HeadBranch receives auxiliary supervision focused on background regions, while the TailBranch employs multi-scale deep supervision via spatial weight maps. This approach enforces functional specialization between branches and mitigates class imbalance.
4. State-of-the-Art Performance: Comprehensive evaluations on the Camelyon16 and Glas benchmark datasets demonstrate that HT-Net outperforms current state-of-the-art models, confirming its effectiveness for histopathological image segmentation.

### 1.1. Related Work

#### 1.1.1. Medical Image Segmentation Methods

The rapid evolution of deep learning has catalyzed transformative advancements in medical image segmentation. U-Net [10] remains a seminal contribution to this field, establishing the groundwork for modern medical image segmentation techniques through the introduction of a symmetric encoder–decoder architecture and a skip connection mechanism. Subsequent research has expanded upon this baseline from various perspectives to address specific diagnostic challenges. Zhou et al. [11] proposed UNet++, which outperforms the traditional U-Net in multiple lesion segmentation tasks by reducing the semantic discrepancy between encoders and decoders through layered dense skip connections. Alom et al. [17] developed R2U-Net, integrating residual connections and recurrent convolutions to augment feature extraction efficacy for retinal vascular and skin lesion segmentation. To facilitate multi-scale feature fusion, Huang et al. [18] introduced UNet3+, utilizing full-scale skip connections and deep supervision techniques to improve contour delineation in organ CT segmentation. Furthermore, Ibtehaz et al. [19] proposed MultiResUNet, which reconstructs convolutional pathways using multi-resolution blocks and residual paths to bridge the semantic gap across adjacent scales, demonstrating robust generalization in multimodal biomedical tasks.

In recent years, Transformers have exhibited significant efficacy in medical image segmentation due to their global self-attention mechanism. Chen et al. [13] introduced TransUNet, which integrates a Vision Transformer into the bottleneck layer of a U-shaped architecture, substantially surpassing convolutional CNN-based approaches in abdominal multi-organ CT segmentation. Similarly, Cao et al. [14] proposed Swin-UNet, a U-shaped architecture composed entirely of Transformers, which attained superior accuracy on the Synapse dataset, particularly for small-scale organs like the pancreas. To address regions with indistinct boundaries, Lin et al. [20] presented DS-TransUNet, which incorporates a dual-scale Swin Transformer encoder to enhance segmentation precision.

Despite these significant advancements, existing methodologies typically rely on a single-branch encoder architecture. These models apply a uniform feature extraction strategy to both Tumor regions and normal background areas, which hinders the model’s ability to learn distinct, region-specific representations. This limitation serves as the fundamental motivation for the dual-branch encoder architecture proposed in this study.

#### 1.1.2. Medical Image Segmentation Based on Attention Mechanisms

Attention mechanisms have emerged as a fundamental technology for improving the feature representation capabilities of medical image segmentation networks. Hu et al. [15] introduced SE-Net, which models inter-channel dependencies via “squeeze-and-excitation” operations that adaptively recalibrate channel-wise feature responses. Although this method efficiently suppresses extraneous textures and enriches the semantic representation of lesions—particularly in dermoscopic image segmentation. However, it exclusively emphasizes global information at the channel level and fails to recognize the spatial localization of lesions. To bridge this gap, Woo et al. [16] proposed CBAM, which progressively integrates channel attention with spatial attention. In brain tumor MRI segmentation, this enables the network to mitigate interference from normal tissue and concentrate specifically on tumor locations, facilitating the dual adaptive learning of feature selection and spatial attention.

Further advancements include Attention U-Net [12], which incorporates attention gating with skip connections. In pancreatic CT segmentation tasks, this efficiently mitigates abdominal background noise and attains enhanced localization precision for target structures with intricate morphology and indistinct borders. Similarly, Roy et al. [21] introduced the scSE module, which concurrently recalibrates features across both spatial and channel dimensions. Their work underscores the synergistic advantages of dual-dimensional attention across diverse benchmarks, including prostate and brain structural MRI segmentation. Cheng et al. [22] subsequently presented ResGANet, utilizing a residual group attention mechanism to concurrently model feature responses, demonstrating the efficacy of combining residual learning with attention strategies.

Recent research has also integrated Transformers to capture long-range dependencies. Chen et al. [23] introduced TransAttUNet, which integrates Transformer self-attention and global spatial attention within a U-shaped architecture, consistently outperforming existing methods in cross-modal tasks such as MRI and multi-organ CT segmentation. AlQurri et al. [24] further enhanced the U-Net architecture by incorporating three-layer attention modules with the Transformer, leveraging the interplay between decoder-side attention and dense skip connections. Moreover, Hussain et al. [25] introduced DCSSGA-UNet, a triadic mechanism encompassing channel, spatial, and semantic-guided attention to optimize multi-class biomedical image segmentation. In the specific context of breast cancer diagnosis, Xi et al. [26] demonstrated the efficacy of Transformer-based external attention, while Li et al. [27] utilized the hybrid architecture to confirm the robustness of Transformer-convolutional feature fusion for histopathology images.

Despite these advancements, current methodologies exhibit significant limitations: (1) standard channel attention often inadequately localizes fine-grained tumor boundaries, (2) conventional spatial attention relies on fixed receptive fields, rendering it unable to accommodate the high variability in tumor size and morphology, and (3) the simple linear superposition of channel and spatial attention fails to fully exploit their potential synergy. These shortcomings provide the fundamental motivation for the Hybrid Multi-Scale Attention (HMSA) module developed in this study.

#### 1.1.3. Medical Image Segmentation Based on Multiscale Feature Extraction

Multiscale feature extraction is a fundamental strategy for addressing target scale variability in medical imaging. The DeepLab series of networks, presented by Chen et al. [28], incorporated Atrous Spatial Pyramid Pooling (ASPP). This method enhances whole-heart segmentation in cardiac CT and MRI by concurrently delineating the intricate contours of cardiac chambers and the larger structures of adjacent major vessels via parallel atrous convolutions, thereby markedly improving the consistency of multiscale object segmentation. Similarly, Yu et al. [29] systematically integrated dilated convolutions into dense prediction tasks. By overlaying convolutional kernels with varying dilation rates to facilitate multi-scale context aggregation, they preserved high recognition accuracy for both capillaries and large arteries in retinal vascular segmentation. To further refine spatial context, Zhao et al. [30] introduced the Pyramid Pooling Module (PPM). In liver CT segmentation tasks, the PPM captures both local edge details and global spatial relationships via multiscale parallel pooling, effectively minimizing inter-organ missegmentation. In the domain of digital pathology, Schmitz et al. [31] employed a multi-scale fully convolutional network for tissue pathology image segmentation, addressing various levels from cell nucleus anomaly detection to comprehensive tissue structure analysis, thereby illustrating the distinct advantages of multiscale techniques in histopathological analysis.

Building on these foundations, recent research has pivoted toward dynamic fusion techniques for multi-scale features. Srivastava et al. [32] introduced MSRF-Net, which utilizes a Dual-Scale Dense Fusion (DSDF) block to facilitate bidirectional transfer of high- and low-resolution features. This approach has demonstrated robust generalization across benchmarks for colorectal polyp, skin lesion, and cell nuclei. Zhang et al. [33] presented SCIF-Net, which integrates a feature pyramid encoder with a multi-scale dynamic aggregation module to tackle substantial scale variations and low-contrast backgrounds in retinal and glandular segmentation. Furthermore, Yuan et al. [34] proposed a U-Net-based context fusion technique that assigns adaptive scale weights to each encoding step via a three-branch feature fusion (TBSFF) block, achieving precise segmentation of colon glands with minimal computational overhead.

Despite these advancements, existing multiscale methodologies are often constrained by static receptive fields and a lack of adaptive cross-scale integration, which hinders the capture of minute pathological features and the alignment of global semantics with fine boundaries. Furthermore, the decoupling of multiscale extraction and attention mechanisms limits the consistent enhancement of discriminative features. To address these deficiencies, we propose the Hybrid Multi-Scale Attention (HMSA) module, which synergistically integrates multiscale convolutions, dilated kernels, and adaptive attention within a unified framework.

## 2. Materials and Methods

### 2.1. Overall Model Architecture

**Figure 1:**
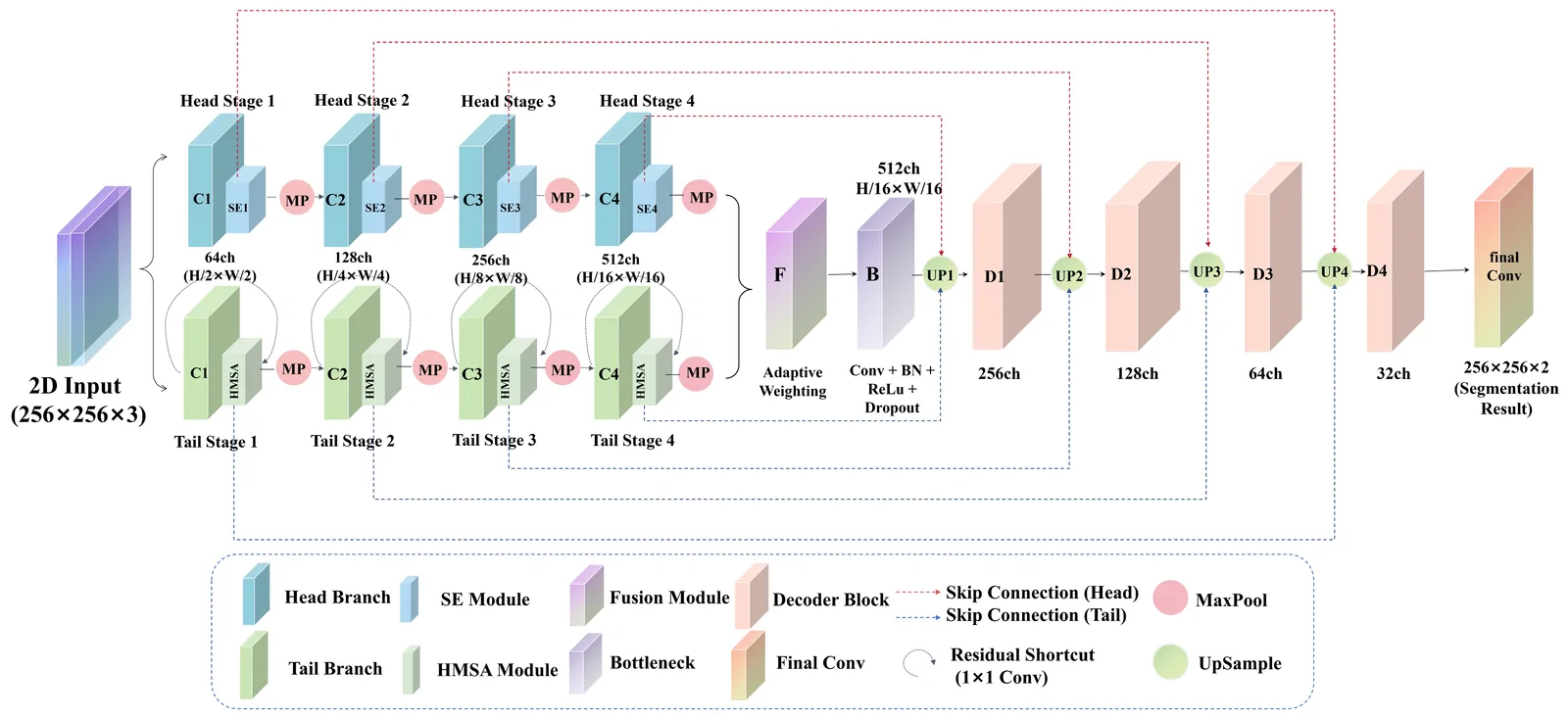
The Architecture of HT-Net.

This research introduces HT-Net, a novel histopathological image segmentation network designed with a dual-branch parallel encoder architecture. HT-Net is specifically engineered to address three critical challenges in breast cancer pathology: feature heterogeneity, class imbalance, and boundary ambiguity. The comprehensive framework is illustrated in Figure 1. The network processes 256 × 256 × 3 RGB histopathological image patches as input and generates a 256 × 256 × 2 segmentation map (differentiating tumor from background). The architecture follows a three-stage encoder-fusion-decoder paradigm:

1. Dual-Branch Encoder: The encoder features a parallel architecture comprising a HeadBranch and a TailBranch. These branches are specialized to perform distinct feature extraction for the background and tumor regions, respectively. Subsequently, a fusion module integrates the bottleneck features from both branches using a spatially adaptive weighting strategy.
2. Symmetrical Decoder: The decoder employs a U-Net-style symmetric architecture, utilizing skip connections to concurrently aggregate multi-scale information from both encoder branches and incrementally restoring spatial resolution to produce the final pixel-level prediction.

### 2.2. Dual-Branch Encoder

The dual-branch encoder consists of the HeadBranch and the TailBranch. While both branches process the same input image, they are equipped with distinct attention strategies tailored to their respective functional roles. Each branch follows a four-stage encoding hierarchy with channel depths of 64, 128, 256, and 512, respectively, while spatial resolution is progressively reduced using max-pooling operations.

#### 2.2.1. HeadBranch Encoder

The Head Branch integrates a Squeeze-and-Excitation (SE) module to emphasize adaptive feature selection within the channel dimension. Each of its four encoding stages consists of a convolutional block, an SE module, and a max-pooling layer. The convolutional block is composed of two 3 × 3 convolutional layers, each followed by batch normalization and a ReLU activation function. The detailed architecture specifications of the HeadBranch Encoder are provided in Table 1.

The SE adaptively recalibrates channel-wise feature responses by explicitly modeling inter-channel dependencies. Specifically, for an input feature map *F* ∈ *R^C^*^×^*^H^*^×^*^W^*, the SE module first compresses the spatial dimension into a channel descriptor *z* ∈ *R^C^* via global average pooling, as shown in Equation (1):

(1) $$z_{c}=\frac{1}{H\times W}\Sigma_{i=1}^{H}\Sigma_{j=1}^{W}F_{c}\left(i,j\right)$$

where *z_c_* denotes the *c*th element of the channel descriptor. Subsequently, two fully connected layers are used to learn the nonlinear relationships between channels, and the Sigmoid function is applied to generate the channel attention weights *s* ∈ *R^C^*, as shown in Equation (2):

(2) *s* = *σ*(*W*^2^ · *δ*(*W*^1^ · *z*))

where $W_{1}\in R^{\frac{C}{r}\times C}$ and $W_{2}\in R^{C\times \frac{C}{r}}$ are learnable parameters, *δ*(·) denotes the ReLU function, *σ*(·) denotes the Sigmoid function, and *r* is the scaling factor (set to 16 in this study). The final output is the channel-weighted feature map, calculated as shown in (3):

(3) $$\tilde{\tilde{F}}=s⊙F$$

where ⊙ denotes element-wise multiplication. The number of output channels in the four encoding stages is 64, 128, 256, and 512, respectively. Following the final encoding stage, an auxiliary segmentation head is added to supervise the background region; this head consists of two convolutional layers and outputs a single-channel background probability map.

#### 2.2.2. TailBranch Encoder

**Figure 2:**
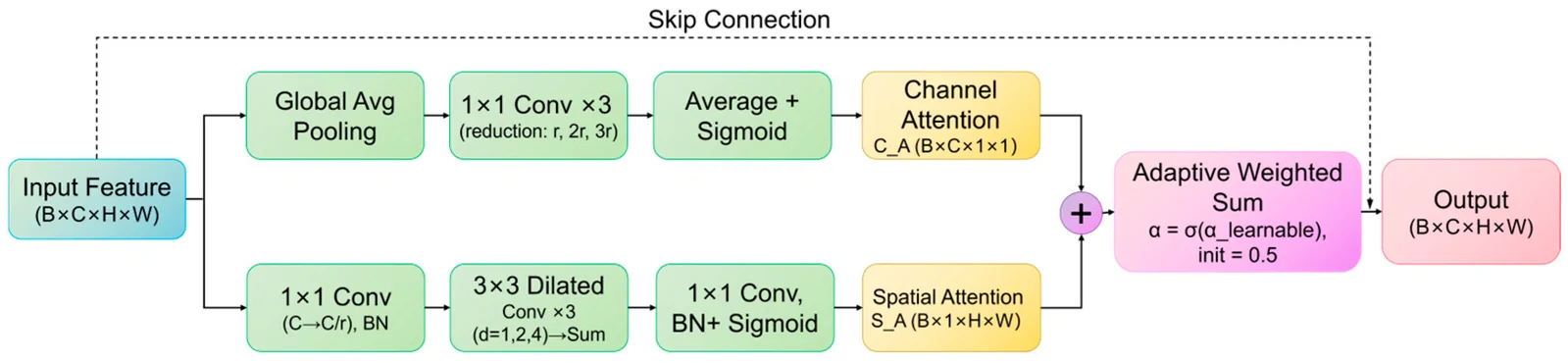
Architecture of the Hybrid Multi-Scale Attention (HMSA). Arrows indicate the direction of feature flow, while different colors distinguish the functional components of the module.

Similarly, the TailBranch comprises four encoding stages, substituting the SE module with the proposed Hybrid Multi-Scale Attention (HMSA) module. This module integrates a multi-scale channel attention branch with a multi-scale spatial attention branch to more effectively capture tumor border features, thereby enhancing spatial detail and multi-scale characteristics inherent in tumor regions. The detailed architecture of the HMSA module is shown in Figure 2.

Specifically, the HMSA module consists of two parallel branches: a channel attention branch and a spatial attention branch, whose outputs are adaptively fused via a learnable weight.

The channel attention branch first applies Global Average Pooling to compress the input feature map from B × C × H × W to B × C × 1 × 1. The pooled features are then fed into three parallel bottleneck pathways with reduction ratios of r, 2r, and 3r (r = 16). The hidden width of the *i*-th pathway is max(⌊$\frac{C}{i\cdot r}$)⌋,*C_min_*), where *C_min_* = 8 is a lower bound that prevents the bottleneck from collapsing at shallow stages (without it, ⌊64/48⌋ = 1 at Stage 0). The resulting hidden widths are 8/8/8, 8/8/8, 16/8/8, and 32/16/10 for Stages 0–3, respectively. Consequently, the three pathways differ in width mainly at the deeper stages. Each pathway consists of two successive 1 × 1 convolutions with an intermediate ReLU activation, where the first convolution reduces the channel dimension and the second restores it to C. The outputs of the three pathways are averaged element-wise and passed through a Sigmoid activation function to generate channel attention weights of size B × C × 1 × 1, as formulated in Equation (4):

(4) $$\mathrm{CA}=\sigma (\frac{1}{3}\Sigma_{i=1}^{3}{\mathrm{MLP}}_{i}\left(\mathrm{GAP}\left(F\right)\right))$$

where *GAP*(·) denotes global average pooling, and *MLP_i_* denotes the *i*-th multi-layer perceptron whose hidden width is max(⌊$\frac{C}{i\cdot r}$⌋, *C_min_*). The outputs of the three pathways are averaged before the Sigmoid activation σ is applied once.

The spatial attention branch first reduces the channel dimension from C to C/r via a 1 × 1 convolution followed by Batch Normalization and ReLU activation. The reduced features are fed into three parallel 3 × 3 dilated convolutions with dilation rates of 1, 2, and 4, corresponding to effective receptive fields of 3 × 3, 5 × 5, and 9 × 9, respectively. The outputs are summed element-wise, followed by a 1 × 1 convolution reducing channels to 1, Batch Normalization, and Sigmoid activation, producing spatial attention weights of size B × 1 × H × W, as shown in Equations (5) and (6):

(5) *F_s_* = *Conv*_1×1_(*F*)

(6) *SA* = *σ*(*Conv*_1×1_(*Σ_d_*_∈{1,2,4}_*DilatedConv*_{3×3,_*_d_*_}_(*F_s_*)))

where *DilatedConv*_{3×3,_*_d_*_}_(·) denotes a 3 × 3 dilated convolution with dilation rate *d*.

To adaptively balance the contributions of the two branches, a learnable scalar parameter α is introduced and constrained to (0,1) via Sigmoid. The final output is obtained by element-wise multiplication of the input feature *x* with the fused attention weights (α·*CA* + (1 − *α*)·*SA*), as formulated in Equations (7) and (8):

(7) *HMSA*(*F*) = *F*⊙(*α* · *CA* + (1 − *α*) · *SA*)

(8) *α* = *σ*(*α*_0_)

where *α*_0_ is a learnable scalar initialized to 0.5, so that the initial fusion weight is α = σ (0.5) ≈ 0.62 (a slight initial preference for the channel attention branch); *α*_0_ is then learned during training. The Tail branch incorporates auxiliary segmentation heads at each encoding stage to provide multi-scale deep supervision for tumor regions.

Furthermore, residual skip connections are implemented at each stage to facilitate gradient flow, utilizing 1 × 1 convolution projection in deep architectures. The detailed architecture of the TailBranch is presented in Table 2.

### 2.3. Dual-Branch Feature Fusion Module

The dual-branch feature fusion module integrates the bottleneck features (512 channels, H/16 × W/16) extracted from both the HeadBranch and TailBranch. This study proposes a spatially adaptive fusion technique that enables the network to dynamically adjust the contribution weights of the two branches according to local content. This mechanism ensures that features from the background-focused and tumor-focused branches are integrated contextually. To further refine the fused representation and improve generalization, the module incorporates a Convolution-BN-ReLU-Dropout architecture, which stabilizes the feature distribution and prevents overfitting during training.

First, the features from the two branches are projected to the same dimension via 1 × 1 convolutions, as shown in Equations (9) and (10):

(9) $$F_{\mathrm{head}}^{\mathrm{proj}}={\mathrm{Conv}}_{1\times 1}\left(F_{\mathrm{head}}\right)$$

(10) $$F_{\mathrm{tail}}^{\mathrm{proj}}={\mathrm{Conv}}_{1\times 1}\left(F_{\mathrm{tail}}\right)$$

Next, the projected features are concatenated, and a lightweight network predicts the spatial fusion weights, as shown in Equation (11):

(11) $$W=\mathrm{Softmax}\left({\mathrm{Conv}}_{1\times 1}\left(\delta \left({\mathrm{Conv}}_{1\times 1}\left(\left[F_{\mathrm{head}}^{\mathrm{proj}};F_{\mathrm{tail}}^{\mathrm{proj}}\right]\right)\right)\right)\right)$$

where [;] denotes the concatenation operation, and *W* ∈ *R*^2×^*^H^*^×^*^W^* is a normalized two-channel weight map. The equation for the fused features is shown in (12):

(12) $$F_{\mathrm{fused}}=W_{0}⊙F_{\mathrm{head}}^{\mathrm{proj}}+W_{1}⊙F_{\mathrm{tail}}^{\mathrm{proj}}$$

where *W*_0_ and *W*_1_ represent the first and second channels of *W*, respectively. The fused features are further refined through convolutional blocks before being fed into the decoder.

### 2.4. U-Net Decoder

The decoder utilizes a U-Net-style symmetric architecture consisting of four distinct stages. At each stage, the feature map resolution is first doubled via transposed convolutions. These upsampled features are then concatenated with the corresponding feature maps from both the HeadBranch and TailBranch via skip connections. This multi-branch integration allows the decoder to recover spatial details while leveraging the specialized features extracted by the dual-branch encoder.

The channel depth is progressively reduced across the four stages to 256, 128, 64, and 32, respectively. Finally, a 1 × 1 convolution layer projects these features into a 2-channel segmentation map (representing background and tumor classes). The decoding process at each stage is formally defined in Equation (13):

(13) $$D_{i}=\mathrm{ConvBlock}\left(\left[\mathrm{UpConv}\left(D_{i+1}\right);F_{i}^{\mathrm{head}};F_{i}^{\mathrm{tail}}\right]\right)$$

where *UpConv*(·) denotes a 2× transposed convolution, *ConvBlock*(·) represents the convolution block, and $F_{i}^{\mathrm{head}}$ and $F_{i}^{\mathrm{tail}}$ represent the features from the *i*-th stage in the Headbranch and Tailbranch, respectively. The number of output channels for the four decoding stages is 256, 128, 64, and 32, respectively. The detailed architecture of the decoder is shown in Table 3.

### 2.5. Division-Based Supervision Loss Function

Deep supervision offers supplementary gradient signals to shallow layers through intermediate classifiers, hence mitigating the vanishing gradient issue in deep networks. Originally proposed by Lee et al. [35], this concept has been widely adopted in medical image segmentation. For instance, Dou et al. [36] integrated deep supervision into 3D medical imaging, while Zhou et al. [11] employed it in UNet++ to facilitate collaborative learning of multi-scale features.

In this study, we develop a task-division supervision loss function tailored to the unique characteristics of our dual-branch architecture. By delivering specific supervisory signals, this function directs the HeadBranch to focus on background recognition and the TailBranch to prioritize tumor detection, thereby fostering effective functional specialization and collaboration. The total objective function, HybridLoss, consists of a primary segmentation loss and task-specific auxiliary losses.

#### 2.5.1. Main Segmentation Loss

To ensure a fair comparison with baseline models, the main segmentation loss uses weighted cross-entropy loss, as defined in Equation (14):

(14) $$L_{\mathrm{main}}=-\Sigma_{c\in \left\{0,1\right\}}{w_{c}}_{\Sigma_{i}g_{c,i}\log\left(p_{c,i}\right)}$$

where *w_c_* represents class weights. We set *w*_0_ = 1.0 for the background, and *w*_1_ = 5.0 for the tumor class to mitigate the impact of class imbalance.

#### 2.5.2. Head Auxiliary Loss

The Head auxiliary loss is designed to steer the HeadBranch toward the accurate representation of background regions. We compute the binary cross-entropy over all pixels of the auxiliary output, using the background mask as the target, as shown in Equation (15):

(15) $$L_{\mathrm{head}}=\frac{1}{N}\Sigma_{i,j}\mathrm{BCE}\left(P_{\mathrm{head}}^{\left(i,j\right)},M_{\mathrm{bg}}^{\left(i,j\right)}\right)$$

where *M_bg_* = 1 [target < 0.5] denotes the background mask (1 for background, 0 for tumor), and *N* is the number of pixels of the auxiliary output. Because the loss is evaluated on both background and tumor pixels, a constant output cannot drive it to zero; reducing it below the level of a constant prediction requires the HeadBranch to spatially discriminate background from tumor.

#### 2.5.3. Tail Auxiliary Loss

The Tail auxiliary loss employs a multiscale deep supervision strategy to guide the TailBranch in learning tumor region features at each encoding stage. An auxiliary head is attached to the output of each TailBranch stage (before max pooling), and the loss weights *w_i_* are assigned as follows: Stage 0 (output resolution H, *w*_0_ = 0.4), Stage 1 (H/2, *w*_1_ = 0.3), Stage 2 (H/4, *w*_2_), and Stage 3 (H/8, *w*_3_ = 0.1), where H denotes the input resolution (i.e., 256, 128, 64, and 32 pixels for a 256 × 256 input). Larger weights are thus given to the shallow, high-resolution stages to emphasize boundary details. No auxiliary head is attached to the H/16 bottleneck. The loss for each scale is calculated by Equation (16):

(16) $$L_{\mathrm{tail}}^{i}=w_{i}\cdot \frac{\Sigma_{j,k}\mathrm{BCE}\left(P_{\mathrm{tail}}^{i,\left(j,k\right)},M_{\mathrm{tumor}}^{\left(j,k\right)}\;\right)\cdot W_{\mathrm{map}}^{\left(j,k\right)}}{\Sigma_{j,k}W_{\mathrm{map}}^{\left(j,k\right)}}$$

where *i* = 0, …, 3 denotes the stage index, and where $M_{\mathrm{tumor}}^{\left(j,k\right)}$ is the binary label of the true tumor mask downsampled to the resolution of stage *i* by max pooling, so that a coarse cell is labeled as tumor whenever it contains at least one tumor pixel. Interpolation-based downsampling (bilinear or nearest-neighbor) of a binary mask is unreliable for small lesions: bilinear interpolation dilutes a lesion into fractional values, and nearest-neighbor sampling can skip it entirely. Max pooling avoids this, at the cost of slightly enlarging the target at coarse scales, which is acceptable because these heads serve only as auxiliary supervision and the coarsest scale has the smallest weight. *W_map_* is the spatial weight map used to emphasize the learning of tumor regions, as shown in Equation (17):

(17) $$W_{\mathrm{map}}^{\left(j,k\right)}=\left\{\begin{array}{c} 5.0\;\mathrm{if}\;\;M_{\mathrm{tumor}}^{\left(j,k\right)}=1\\ 0.5\;\mathrm{if}\;\;M_{\mathrm{tumor}}^{\left(j,k\right)}=0 \end{array}\right.$$

#### 2.5.4. Total Loss Function

The total loss function integrates these components to fully leverage the dual-branch architecture while maintaining comparability with existing methods, as shown in (18):

(18) $$L_{\mathrm{total}}=L_{\mathrm{main}}+\lambda_{\mathrm{head}}\cdot L_{\mathrm{head}}+\lambda_{\mathrm{tail}}\cdot \Sigma_{i=0}^{3}L_{\mathrm{tail}}^{i}$$

where *λ_head_* = 0.3, and *λ_tail_* = 0.4. This hybrid loss design ensures that HT-Net benefits from specialized branch supervision without deviating from standard performance benchmarks.

### 2.6. Datasets

#### 2.6.1. Camelyon16 Dataset

The Camelyon16 dataset [37] is a widely recognized benchmark for histopathological image analysis. Co-released by Radboud University Medical Center and the University Medical Center Utrecht in the Netherlands, it is designed to assess algorithms for segmenting lymph node metastases in breast cancer. The dataset consists of 400 H&E-stained whole-slide images (WSIs), comprising 270 slides in the training set (160 normal slides and 110 tumor-infected slides) and 130 slides in the test set. Each tumor-positive slide is provided with ground truth annotations in XML format, delineating metastatic regions as defined by expert pathologists. This study utilizes this dataset as the principal evaluation benchmark.

#### 2.6.2. Glas Dataset

The Glas (Gland Segmentation in Colon Histology Images Challenge Contest) dataset [38] serves as a definitive standard in colon gland segmentation, collaboratively published by Sirinukunwattana et al. It consists of 165 microscopic images obtained from 16 H&E-stained colon tissue slices. The data is partitioned into a training set of 85 images (comprising both benign and malignant glands) and a test set of 80 images (60 in TestA and 20 in TestB). The dataset is characterized by diverse glandular morphologies, irregular boundaries, and complex lumen structures, often with neighboring glands in close proximity. We employ the Glas dataset as a secondary evaluation benchmark to assess the generalization capability of HT-Net in the context of colon glandular segmentation.

#### 2.6.3. Data Preprocessing Method

Given that WSIs typically contain billions of pixels, they cannot be processed directly by deep learning models. This study utilizes a sliding window approach to extract fixed-size patches followed by rigorous quality control and mask generation. The specific steps are detailed as follows:

Step 1. Patch Extraction: The Level 0 pixel size of the slides is approximately 0.243 µm (Radboud University Medical Center) and 0.226 µm (University Medical Center Utrecht), equivalent to about 40× magnification. Patches were extracted at Level 1 of the pyramid, which is downsampled by a factor of 4 from Level 0 (approximately 10×, 0.90–0.97 µm/pixel), using non-overlapping sliding windows with a stride of 256 pixels, so that each 256 × 256 patch covers about 0.23–0.25 mm of tissue. To filter out non-informative areas, a tissue threshold of 70% was applied; only patches containing at least 70% tissue were retained.

Step 2. Quality Control: To ensure the integrity of the training data, we performed a multi-dimensional screening process based on six criteria. (1) Patches with a pixel standard deviation below 15 were removed as low-contrast or solid-color; (2) Underexposed (mean brightness < 30) or overexposed (mean brightness > 245) patches were excluded; (3) Patches with less than 25% tissue area (mostly background) were discarded; (4) Blurry or out-of-focus images with a Laplacian variance below 80 were eliminated; (5) Faded or grayscale-like patches with a saturation score below 10 were removed; (6) WSI edge artifacts containing black regions exceeding 15% were discarded. Significantly, image patches with tumor annotations were preserved regardless of these thresholds to prevent the loss of critical positive samples. In total, approximately 65% of the patches extracted in Step 1 were discarded by the above screening, and the remaining 140,040 patches were used to build the final dataset.

Step 3. Dataset Partitioning: A stratified random splitting approach was employed to partition the data into training, validation, and test sets in a 6:2:2 ratio, maintaining uniform positive-to-negative sample ratios across all subsets. This resulted in a final dataset of 140,040 patches (84,024 for training, 28,008 for validation, and 28,008 for testing).

Step 4. Segmentation Mask Generation: Binary masks were generated for each patch based on the polygon coordinates in the XML annotation. Vertex coordinates were first converted from the Level 0 to Level 1. Subsequently, we utilized OpenCV’s (version 4.8.1) fillPoly function to create 256 × 256 binary masks, in which tumor regions were assigned a pixel value of 255 and background region a value of 0.

### 2.7. Experimental Setup

#### 2.7.1. Evaluation Metrics

Segmentation performance was evaluated with four standard pixel-wise metrics: mean Intersection over Union (mIoU), mean Dice coefficient (mDice), mean Recall (mRecall), and mean Precision (mPrecision), whose definitions follow common practice in medical image segmentation [39]. For each class, the metrics are computed from the pixel-level true positives (TP), false positives (FP), and false negatives (FN), and then averaged over the K = 2 classes (background and tumor for Camelyon16; background and gland for GlaS).

#### 2.7.2. Implementation Details

To validate the efficacy of the proposed HT-Net and ensure a fair comparison with state-of-the-art methodologies, all models were trained under uniform experimental conditions. The primary objective function utilized was a weighted cross-entropy loss, with a weight of 5.0 assigned to the tumor class to address sample imbalance.

Network optimization was performed using the AdamW optimizer, with hyperparameters set to β_1_ = 0.9, β_2_ = 0.999, and a weight decay of 1 × 10^−3^. The initial learning rate was established at 1 × 10^−4^, employing a cosine-smoothed annealing technique for adjustment, with a minimum learning rate of 1 × 10^−6^. For the Camelyon16 dataset, the batch size was set to 16 with a maximum of 100 epochs. To ensure training stability, gradient clipping (maximum norm of 1.0) was applied, and an early stopping criterion was implemented to terminate training after 15 epochs without enhancement. Automatic Mixed Precision (AMP) was enabled to accelerate training and optimize GPU memory utilization. For the Glas dataset, the batch size was adjusted to 8 due to the reduced image dimensions, the maximum epochs were increased to 400, and the early stopping criterion was established to terminate training after 50 epochs without improvement.

Prior to being fed into the network, all images were scaled to 256 × 256 pixels (Camelyon16 patches were extracted at this size and required no resizing; GlaS images were resized from their original size of approximately 775 × 522 pixels). The input size of 256 × 256 was chosen as a compromise between the field of view (about 0.25 mm at Level 1 for Camelyon16), computational cost, and GPU memory, and because it is divisible by the 2^4^ downsampling required by the four-stage encoder. It was not tuned as a hyperparameter, and the sensitivity of HT-Net to patch size and resolution was not evaluated. Data augmentation strategies were employed to improve model robustness, including random rotation (angles randomly chosen from {0°, 90°, 180°, 270°}), random horizontal and vertical flipping with a chance of 0.5, and random color jittering (with a variation range of 0.5 for brightness, contrast, and saturation, and 0.05 for hue). All input patches were normalized utilizing ImageNet pre-training parameters (mean [0.485, 0.456, 0.406], standard deviation [0.229, 0.224, 0.225]).

For each model, the best epoch on each validation fold was selected, and results are reported as the mean ± standard deviation over the five folds, using identical fold assignments for all models. Because the training sets of different folds overlap, fold-level scores are not independent; therefore, formal significance tests were not performed. All experiments were conducted on a workstation equipped with an NVIDIA RTX 4090 GPU (NVIDIA Corporation, Santa Clara, CA, USA) with 24 GB VRAM. Computational complexity (parameters, GFLOPs, inference latency, and GPU memory) was measured on the same GPU with a 256 × 256 input for all models.

## 3. Results

### 3.1. Results on the Camelyon16 Dataset

To rigorously evaluate the segmentation performance of HT-Net, we selected five state-of-the-art approaches as comparative baselines. These models represent the most prominent technical paradigms in current medical image segmentation:

1. U-Net [10] and UNet++ [11]: These serve as the primary convolutional benchmarks. U-Net establishes the baseline for standard encoder–decoder architectures, while UNet++ represents the advancement of nested dense connections.
2. UCTransNet [40]: This model incorporates a channel Transformer with the skip connections, representing a significant evolution attention-augmented U-shaped networks.
3. VM-UNet [41]: Based on the Visual State Space Model (Mamba), this framework illustrates the recent application of innovative sequence modeling paradigms in the medical imaging domain.
4. nnWNet [42]: Integrating the WNet architecture into the nnUNet automated training framework, this model facilitates continuous feature propagation through the parallel design of local convolutional branches and global Transformer branches. It is currently one of the most competitive methods in terms of overall performance.

By encompassing pure convolutional networks, attention-enhanced models, state-space models, and hybrid Transformer-CNN architectures, these five methodologies provide a multidimensional benchmark. This selection ensures a comprehensive assessment of HT-Net’s efficacy against the diverse technical strategies currently dominating the field.

Table 4 displays the quantitative comparison results between HT-Net and state-of-the-art methodologies on the Camelyon16 dataset. HT-Net achieved superior performance across all primary metrics, with a mIoU of 82.05% and a mDice of 89.37%, thereby substantiating the efficacy of the proposed architecture.

HT-Net outperformed classical convolutional networks, demonstrating improvements of 2.30 and 2.49 percentage points in mDice relative to U-Net and UNet++, respectively. Correspondingly, it showed gains of 2.77 and 3.07 percentage points in mIoU. These results suggest that the dual-branch, “division-of-labor” strategy provides a significant advantage in distinguishing between complex tumor features and background characteristics.

Compared to the attention-augmented UCTransNet [40], HT-Net showed enhancements of 1.62 and 2.12 percentage points in mDice and mIoU, respectively. This indicates that the dual-branch differential attention mechanism possesses superior regional discrimination capabilities compared to a single-branch channel Transformer. Furthermore, HT-Net surpassed the state-space-based VM-UNet [41] by 1.30 and 1.94 percentage points; the multiscale dilated convolutions within the HMSA module effectively address the inherent limitations of SSM-based approaches in capturing local boundary details.

Finally, in comparison to nnWNet [42], which showed the most competitive baseline performance, HT-Net maintained a lead of 0.86 and 1.08 percentage points in mDice and mIoU. The implementation of a task-specific supervision loss function further refines the functional specialization of the dual branches, allowing the model to sustain high detection sensitivity for tumorous regions while preserving background integrity.

**Figure 3:**
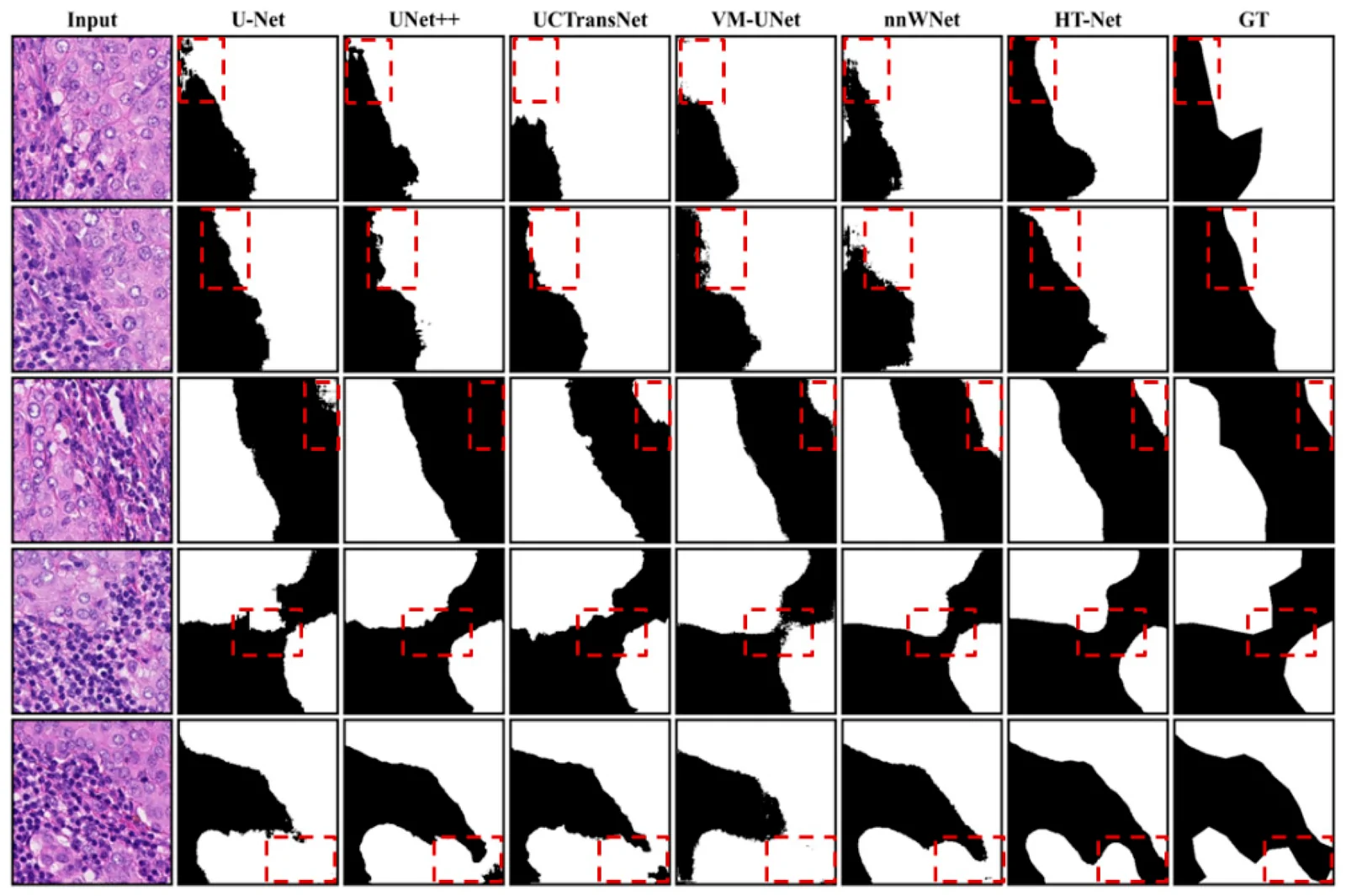
Visual Comparison of segmentation results between HT-Net and baseline methods on the Camelyon16 test set. Regions highlighted by the dashed boxes are for better visualization of the prediction differences. GT represents the ground truth.

Figure 3 provides a visual comparison of segmentation results between HT-Net and baseline methods on the Camelyon16 test set. Each row depicts a challenging histopathological image patch, while columns represent, from left to right: the input image, the prediction of baseline methods, HT-Net prediction, and the ground truth (GT). Overall, HT-Net’s predictions outperform the baseline models in both morphological integrity and boundary precision.

In instances involving large tumorous regions (Rows 1 and 2), both U-Net and UNet++ exhibit varying degrees of under-segmentation characterized by irregular edges. In contrast, HT-Net effectively preserves the primary tumor morphology, yielding smoother boundaries that closely align with the ground truth. In samples with distinct tumor-background delineations (Rows 3 and 4), while all three methodologies correctly identify the core tumor areas, U-Net and UNet++ show significant misclassifications and boundary artifacts. By leveraging the multiscale spatial attention features of its HMSA module, HT-Net achieves superior precision and clarity in delineating these transition zones.

For the most difficult cases, where clinical characteristics are intricate and local textures closely resemble tumor tissue (Rows 5 and 6), U-Net produces a substantial amount of false-positive background noise. Although UNet++ offers some improvement, isolated artifacts remain evident. Conversely, HT-Net effectively mitigates background interference through its task-specific supervision strategy, maintaining a robust focus on actual tumor locations. These visualization findings corroborate the quantitative metrics, further affirming the superiority of HT-Net in histopathological tumor segmentation.

### 3.2. Results on the Glas Dataset

We employed five same baseline methods to further assess the generalization capabilities of HT-Net on the Glas colon gland segmentation dataset.

The quantitative results conclusively demonstrate the efficacy and generalization capabilities of the proposed strategy for the Glas colon gland segmentation challenge. Table 5 presents a performance comparison between HT-Net and leading state-of-the-art approaches on the Glas dataset. HT-Net achieved superior performance, reaching 93.04% mDice and 86.95 mIoU, thereby confirming its effectiveness in the context of colon adenocarcinoma segmentation.

HT-Net outperformed classical convolutional networks, demonstrating gains of 0.85 and 1.38 percentage points in mDice, and 1.43 and 2.36 percentage points in mIoU compared to U-Net and UNet++, respectively. These results indicate that the dual-branch collaborative strategy provides a substantial advantage in reconciling the morphological disparities between glandular structures and stromal characteristics.

Compared to the attention-augmented UCTransNet [40], HT-Net showed enhancements of 1.01 and 1.69 percentage points in mDice and mIoU, respectively. This signifies that the dual-branch differential attention mechanism possesses superior regional discrimination capabilities compared to single-branch Transformer architectures. Furthermore, HT-Net surpassed the state-space-based VM-UNet [41] by 0.53 and 0.88 percentage points. The multiscale dilated convolutions within the HMSA module effectively address the limitations of SSM-based approaches in resolving fine features at glandular boundaries.

Finally, in comparison to nnWNet [42], which showed the most competitive performance, HT-Net maintained an advantage of 0.30 and 0.47 percentage points in mDice and mIoU. The task-specific supervision loss function further enhances the functional specialization of the dual branches, enabling the model to achieve higher detection accuracy for glandular regions while preserving the integrity of the stromal background.

**Figure 4:**
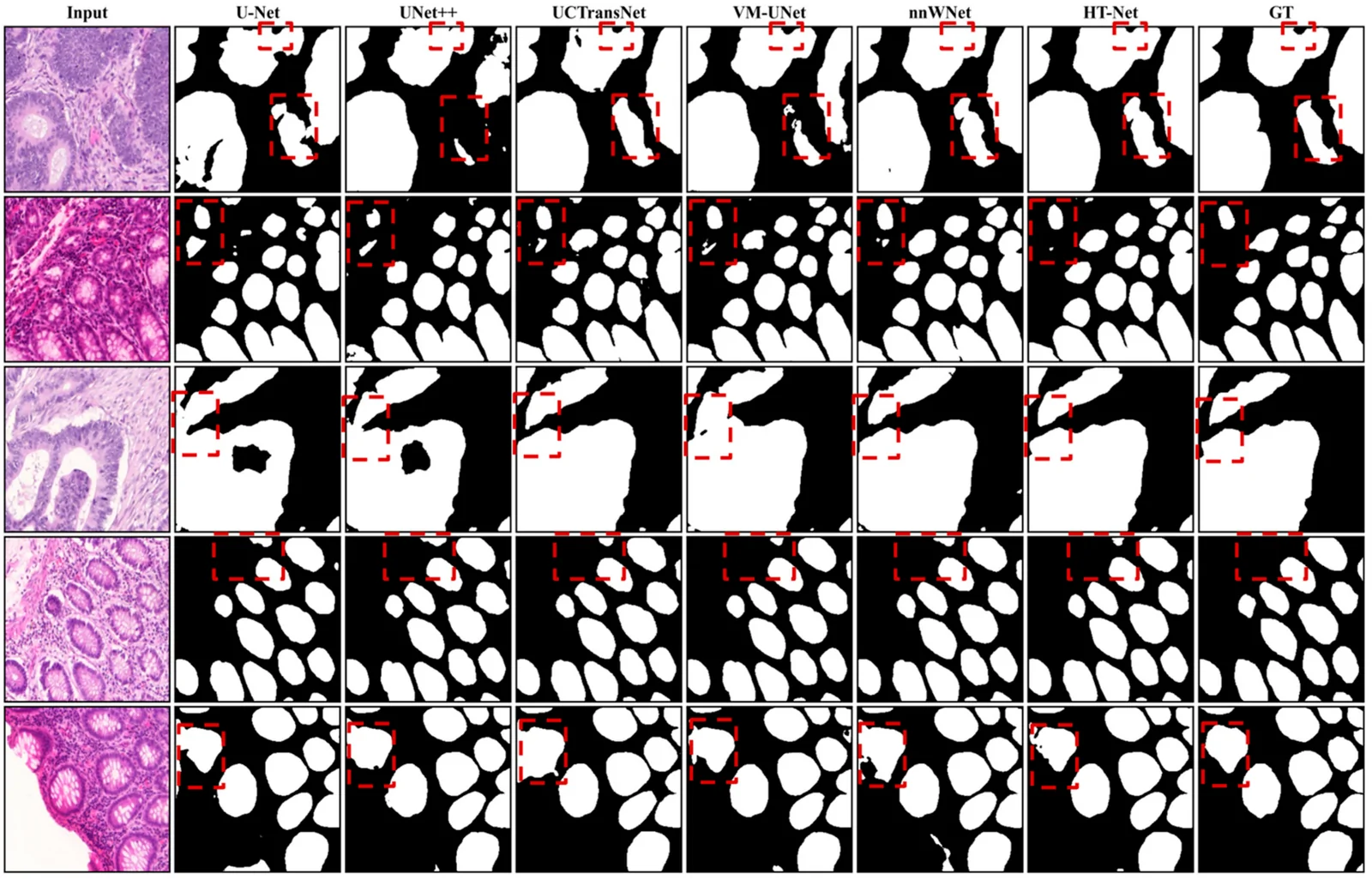
Visual Comparison of segmentation results between HT-Net and baseline methods on the Glas test set. Regions highlighted by the dashed boxes are for better visualization of the prediction differences. GT represents the ground truth.

Figure 4 presents a visual comparison of segmentation results between HT-Net and baseline methods on the Glas test set. Each row depicts a representative challenging image patch, while the columns display, from left to right: the input image, the prediction of baseline methods, HT-Net prediction, and the ground truth (GT). Overall, HT-Net’s predictions outperform the baseline models in both morphological integrity and boundary precision.

In instances involving large glandular regions (Rows 1 and 2), both U-Net and UNet++ exhibit varying degrees of omission characterized by irregular edges. Conversely, HT-Net effectively preserves the primary glandular morphology, generating smoother boundaries that align closely with the ground truth. In samples with distinct tissue delineations (Rows 3 and 4), while all three methodologies successfully identify the core glandular areas, U-Net and UNet++ show significant misclassifications and under-segmentation at the boundary transitions. By leveraging the multiscale spatial attention features of its HMSA module, HT-Net achieves enhanced precision and clarity in delineating these complex boundary regions.

For difficult cases with intricate clinical features where local textures closely resemble glandular tissue (Rows 5 and 6), U-Net generates a substantial number of false-positive background artifacts. Although UNet++ offers relative improvements, isolated noise sites remain prevalent. In contrast, HT-Net efficiently mitigates stromal background interference through its task-specific supervision strategy, maintaining a robust focus on the actual target locations. These visualization findings corroborate the quantitative metrics, further affirming the superiority of HT-Net for segmentation tasks in histopathological imaging.

### 3.3. Ablation Study

We conducted progressive ablation experiments on the Camelyon16 dataset to evaluate the individual contributions of the dual-branch architecture, the HMSA module, and the task-specific supervision strategy to the overall performance of HT-Net. We systematically introduced each component and monitored performance variations to progressively validate their efficacy. The specific configurations are defined as follows:

1. Baseline: A conventional U-Net encoder–decoder architecture, devoid of attention mechanisms or auxiliary supervision. This model establishes a performance lower bound and illustrates the intrinsic constraints of the single-branch homogenous feature extraction methodology.
2. Dual-SE: The single-branch encoder is enhanced into a dual-branch parallel architecture, using SE attention modules in both the HeadBranch and TailBranch. This configuration aims to evaluate the performance enhancements intrinsic to the dual-branch parallel architecture—specifically, whether increasing the number of branches alleviates regional feature heterogeneity when the attention mechanism remains constant.
3. Dual-SE-HMSA: The SE module in the TailBranch is replaced by the proposed HMSA module, while the SE module in the HeadBranch remains unchanged. This variation independently assess the efficacy of the HMSA module, investigating if the incorporation of a differentiated attention mechanism can enhance the multiscale perception of tumor boundary characteristics.
4. HT-Net (Comprehensive Model): This configuration enhances the Dual-SE-HMSA by incorporating a task-specific supervision loss function, which utilizes background-region-targeted auxiliary supervision for the HeadBranch and multiscale tumor-region deep supervision for the TailBranch. This stage determines if the task-specific supervision technique can facilitate effective functional distinction between the two branches at the objective function level, hence optimizing overall segmentation performance.

The four configurations exhibit a clear evolutionary trend: single-branch baseline → dual-branch homogenous attention → dual-branch differentiated attention → dual-branch differentiated attention with task-specific supervision. By altering only one variable at each stage, we ensured the validity of the conclusions derived from this ablation study. Each stage alters only one variable, hence guaranteeing the validity of the conclusions gained from the ablation experiment.

#### 3.3.1. Ablation Results on the Camelyon16

**Figure 5:**
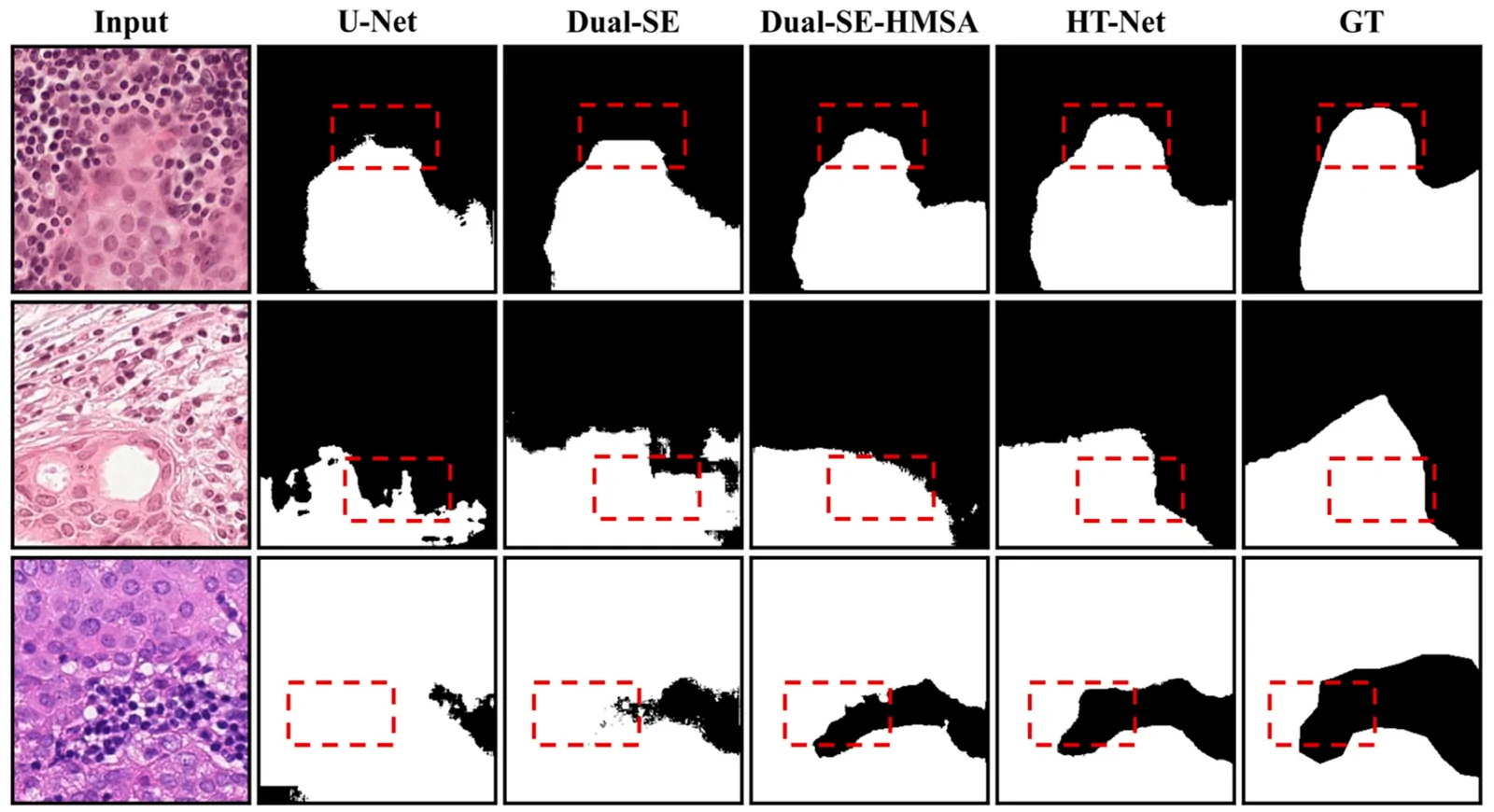
Visual Comparison of segmentation results from ablation experiments on various HT-Net components using the Camelyon16 dataset. Regions highlighted by the dashed boxes are for better visualization of the prediction differences. GT represents the ground truth.

Specifically, the quantitative and visual results of the ablation experiment for HT-Net components on the Camelyon16 dataset are shown in Table 6 and Figure 5, respectively. Table 6 presents the ablation study results on the Camelyon16 dataset, demonstrating the incremental performance gains achieved by each component of the proposed HT-Net. Compared to the baseline U-Net, the integration of the Dual-SE and HMSA modules consistently improved segmentation accuracy across all metrics. Ultimately, the full HT-Net achieved superior performance, reaching 89.39% mDice and 82.06% mIoU, thereby validating the effectiveness of the proposed architectural enhancements for histopathology image analysis.

As illustrated in Figure 5, the Baseline (U-Net) produces considerable “jaggedness” in predicted boundaries, characterized by a high volume of misclassified pixels in the tumor–background transition zones and a failure to identify fine substructures. Following the implementation of the dual-branch architecture (Dual-SE), the continuity of the tumor body is enhanced, and background misclassification rates are reduced; however, irregular noise persists at the boundaries.

By replacing the SE module with the HMSA module (Dual-SE-HMSA), the multiscale dilated convolutions effectively expand the receptive field, yielding smoother and more continuous boundary contours while better preserving substructure integrity. Finally, the incorporation of task-specific auxiliary supervision in the full HT-Net significantly improves background suppression. This configuration achieves the optimal overlap between the predicted tumor regions and the ground truth (GT). These results confirm that our functional specialization strategy—in which the HeadBranch focuses on background representation and the TailBranch prioritizes tumor detection—effectively directs the feature learning of the dual branches to attain peak segmentation performance.

#### 3.3.2. Ablation Results on the Glas Dataset

**Figure 6:**
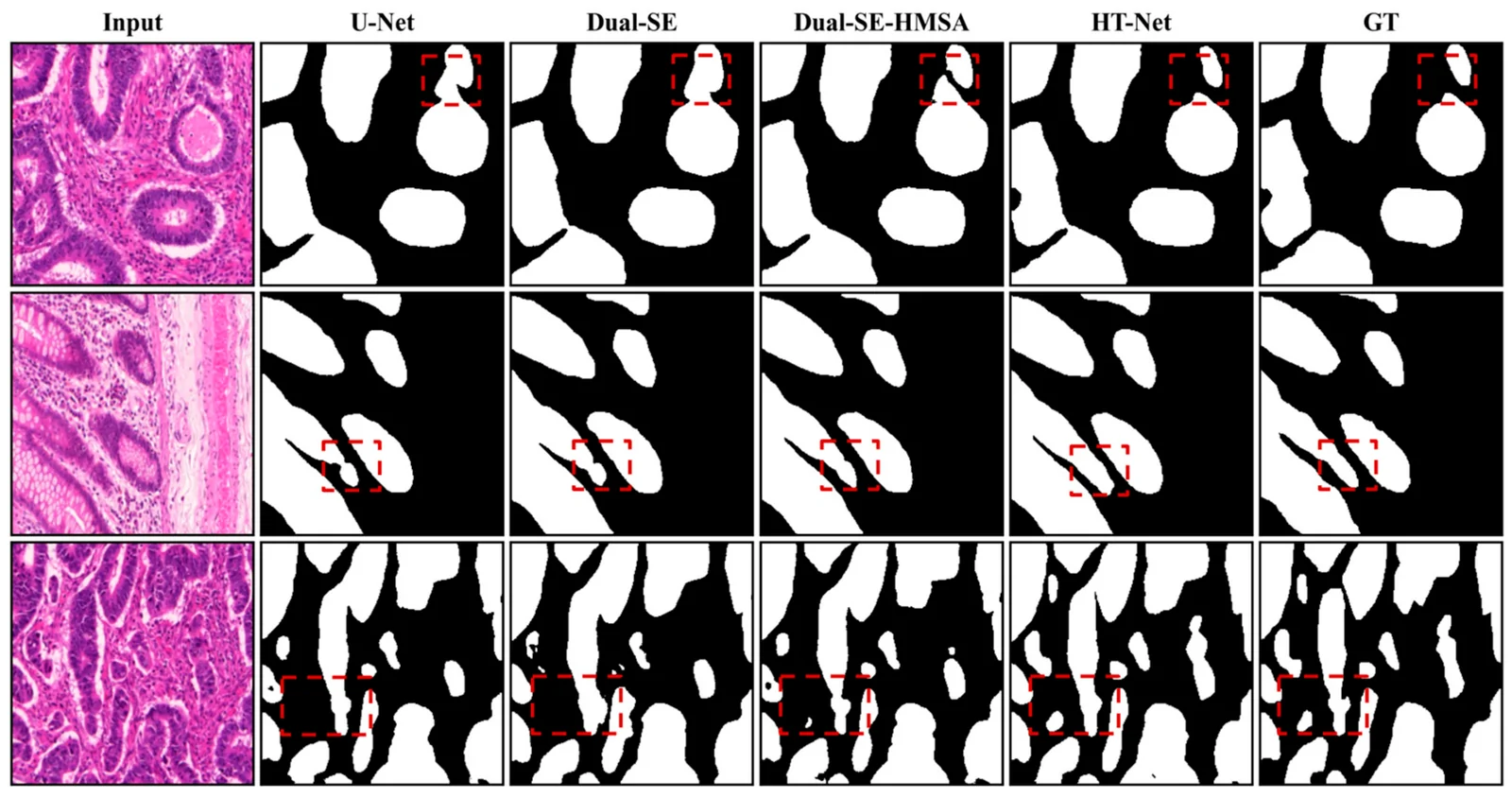
Visual Comparison of segmentation results from ablation experiments on various HT-Net components using the Glas dataset. Regions highlighted by the dashed boxes are for better visualization of the prediction differences. GT represents the ground truth.

Moreover, we performed the ablation study on the Glas dataset and the quantitative and visual results for HT-Net components are shown in Table 7 and Figure 6, respectively. Table 7 summarizes the ablation study results on the Glas dataset, illustrating the individual contributions of the proposed modules to the overall segmentation performance. The baseline U-Net is progressively enhanced by the addition of the Dual-SE and HMSA components, resulting in steady improvements across all evaluated metrics. The complete HT-Net architecture achieves the highest performance, with mDice and mIoU reaching 93.04% and 86.95%, respectively. These results further confirm the robustness and efficacy of the integrated modules in capturing complex morphological features in gland segmentation tasks.

The visualization results depicted in Figure 6 substantiate the quantitative findings: the Baseline (U-Net) generates relatively imprecise representations of glandular boundaries, exhibiting indistinct separations between the glandular lumen and the adjacent stroma. In this configuration, several small glands are either overlooked or incorrectly predicted as merged entities.

Following the implementation of the dual-branch architecture (Dual-SE), the segmentation fidelity of the primary glandular regions is improved, and the incidence of inter-gland fusion is reduced. However, the gains in boundary precision remain limited, with instances of over-segmentation and under-segmentation persisting in complex areas. Replacing the SE module with the HMSA module (Dual-SE-HMSA) markedly improves the model’s capacity to discern glandular contours. By incorporating multiscale receptive fields, this module yields more defined boundaries while better preserving internal glandular structures.

Finally, the introduction of task-specific auxiliary supervision in the full HT-Net further reduces misclassified regions within the stromal background. This approach achieves optimal alignment between the predicted glandular boundaries and the ground truth (GT), producing the most precise overall segmentation results. These findings further substantiate the efficacy of the task-specific supervision strategy in facilitating the functional specialization of dual branches when navigating the diverse morphological variations inherent in glandular segmentation.

## 4. Discussion

Table 8 compares the computational complexity of all models under identical settings. HT-Net has the fewest parameters (24.0 M), and its computational cost (46.3 GFLOPs) is lower than that of U-Net, UNet++, and UCTransNet, but higher than that of nnWNet (18.9 GFLOPs). Its latency (11.4 ms) and GPU memory usage (2625.7 MB) are slightly higher than those of U-Net (8.9 ms, 2285.6 MB) and nnWNet (10.7 ms, 2518.3 MB), and clearly lower than those of UNet++ (18.5 ms, 2901.6 MB) and UCTransNet (15.6 ms, 3427.5 MB). The dual-branch design therefore adds some overhead relative to the lightest baselines, but the overhead is modest in absolute terms: HT-Net processes a 256 × 256 image in 11.4 ms using about 2.6 GB of GPU memory on a single RTX 4090. However, these measurements were obtained on a workstation GPU, and its suitability for resource-constrained clinical deployments still needs to be verified on the target hardware.

HT-Net is intended as a decision-support tool for pathologists assessing sentinel lymph node metastasis, acting as a second reader rather than replacing the pathologist. Following the deployment-oriented descriptions of AI-assisted diagnostic workflows in medical imaging [43], we outline the envisioned pipeline, which has not yet been validated end to end. (1) A scanned whole-slide image (WSI) is loaded and a tissue mask is obtained from a low-resolution thumbnail to exclude the glass background. (2) The slide is tiled at pyramid Level 1 (approximately 10×) into 256 × 256 patches with a stride of 256, the same setting used to build our training data, and patches with less than 70% tissue are skipped. (3) The patches are processed in batches by HT-Net in inference mode, in which only the main segmentation output is computed and the auxiliary heads are not used. (4) The patch predictions are written back to their tile coordinates to form a slide-level mask, which can be mapped to Level 0 coordinates and shown in the slide viewer as a semi-transparent overlay or contour, leaving the final decision to the pathologist. Tile-boundary artifacts can be reduced by using overlapping tiles (e.g., a stride of 128) and averaging the overlapping predictions, at roughly four times the computation.

Regarding resources, HT-Net processes a 256 × 256 patch in 11.4 ms and uses about 2.6 GB of GPU memory on a single RTX 4090 (Table 8). Based on this latency, the pure inference throughput is roughly 88 patches per second, so a slide with 5000 tissue patches would require on the order of one minute of network inference; this estimate excludes slide reading and mask stitching, which were not measured. Because the memory footprint fits a single workstation-grade GPU, the model can be deployed locally next to the slide viewer, avoiding the transfer of gigapixel images to external servers, while a server-based deployment remains an option for batch processing of many slides. We emphasize that our experiments evaluate patch-level segmentation on pre-extracted patches. End-to-end slide-level evaluation, measurement of complete per-slide processing time, integration into clinical viewing software, and prospective validation with pathologists were not performed and are left for future work.

Furthermore, while validated on breast and colonic tissues, the model’s generalizability to other pathological types warrants further investigation. Future research will focus on three primary directions: (1) Model Compression: Implementing knowledge distillation or network pruning to develop a lightweight version of HT-Net suitable for resource-constrained clinical settings without sacrificing accuracy. (2) Domain Expansion: Assessing the framework’s versatility across a broader spectrum of histopathological datasets, with a specific focus on challenging scenarios such as diminutive glands and sparse metastatic distributions. (3) Hyperparameter Optimization: The hyperparameters of HT-Net, such as the auxiliary loss weights, the class weight and the learning rate, were set manually in this study. Automated optimization of these parameters, for example, with evolutionary algorithms such as genetic algorithms [44], is a promising direction for improving performance.

**Table 8:** Computational complexity of HT-Net and the compared methods. All models were measured under identical conditions with a 256 × 256 input on an NVIDIA RTX 4090 GPU (batch size 32, FP32); latency is the average over 100 forward passes after 50 warm-up iterations. N/A GFLOPs of VM-UNet are not reported because its selective-scan operator is implemented as a custom CUDA kernel that the FLOPs profiler used for the other models ([thop/fvcore]) does not count, so the same procedure would give an incomplete value.

Model	Params (M)	GFLOPs	Latency (ms)	GPU Memory (MB)
U-Net	31.0	54.7	8.9	2285.6
UNet++	35.8	137.8	18.5	2901.6
UCTransNet	66.5	50.8	15.6	3427.5
VM-UNet	27.4	N/A	9.3	2387.8
nnWNet	28.4	18.9	10.7	2518.3
HT-Net (ours)	24.0	46.3	11.4	2625.7

## 5. Conclusions

This study introduced HT-Net, a novel dual-branch tumor segmentation network designed to address the critical challenges of feature heterogeneity, class imbalance, and indistinct boundaries in histopathological imaging. By integrating differentiated encoding, multiscale attention, and task-specific supervision, HT-Net achieves a synergistic balance between background suppression and tumor localization. The proposed architecture is conceptually aligned with biomimetic principles of functional specialization, selective attention, and complementary information processing, in which different processing pathways contribute specialized representations that are adaptively integrated for robust perception. Our key contributions are summarized as follows:

1. Dual-Branch Parallel Encoding: We developed a parallel architecture where the HeadBranch (utilizing SE attention) focuses on background feature selection, while the TailBranch (utilizing the proposed HMSA module) targets tumor-specific characteristics. Ablation results confirm that this collaborative feature extraction improves mDice and mIoU by 0.84 and 0.89 percentage points, respectively, over the single-branch baseline.
2. Hybrid Multi-Scale Attention (HMSA): The HMSA module effectively captures multi-granularity channel dependencies and expansive spatial contexts through parallel dilated convolutions (dilation rates: 1, 2, 4). This differentiated attention mechanism outperformed single-scale alternatives, enhancing mDice by 0.83 and mIoU by 1.36 percentage points.
3. Task-Specific Supervision: A specialized loss function was formulated to enforce functional differentiation. By applying background-targeted supervision to the HeadBranch and multiscale deep supervision to the TailBranch, we mitigated class imbalance and optimized inter-branch collaboration, yielding further gains of 0.61 (mDice) and 0.52 (mIoU) percentage points.
4. SOTA Performance and Generalization: Comprehensive evaluations on the Camelyon16 and Glas datasets demonstrate that HT-Net consistently outperforms leading methods, including nnWNet and VM-UNet. Specifically, HT-Net achieved a mDice of 89.37% on Camelyon16 and 93.04% on Glas, underscoring its robustness across different pathological contexts and morphological variations.

## Figures and Tables

**Table 1:** Detailed Architecture of the HeadBranch Encoder.

Stage	Layer Details	Output Channels
0	[Conv3 × 3 + BN + ReLU] × 2, SE, MaxPool2 × 2	64
1	[Conv3 × 3 + BN + ReLU] × 2, SE, MaxPool2 × 2	128
2	[Conv3 × 3 + BN + ReLU] × 2, SE, MaxPool2 × 2	256
3	[Conv3 × 3 + BN + ReLU] × 2, SE, AuxHead, MaxPool2 × 2	512

**Table 2:** Encoder Structure of the TailBranch.

Stage	Layer Details	Output Channels
0	[Conv3 × 3 + BN + ReLU] × 2, HMSA, Skip, AuxHead, MaxPool	64
1	[Conv3 × 3 + BN + ReLU] × 2, HMSA, Skip, AuxHead, MaxPool	128
2	[Conv3 × 3 + BN + ReLU] × 2, HMSA, Skip, AuxHead, MaxPool	256
3	[Conv3 × 3 + BN + ReLU] × 2, HMSA, Skip, AuxHead, MaxPool	512

**Table 3:** Architecture of the U-Net Decoder.

Stage	Layer Details	Output Channels
0	TransConv2 × 2, Concat(Head + Tail), [Conv3 × 3 + BN + ReLU] × 2	256
1	TransConv2 × 2, Concat(Head + Tail), [Conv3 × 3 + BN + ReLU] × 2	128
2	TransConv2 × 2, Concat(Head + Tail), [Conv3 × 3 + BN + ReLU] × 2	64
3	TransConv2 × 2, Concat(Head + Tail), [Conv3 × 3 + BN + ReLU] × 2	32
Output	Conv1 × 1	2

**Table 4:** Comparison of Segmentation Performance Between HT-Net and Baseline Methods on the Camelyon16 Dataset.

Model	mDice	mIoU	mRecall	mPrecision
U-Net	87.09 ± 1.86	79.28 ± 2.31	85.35 ± 2.14	89.44 ± 2.03
UNet++	86.88 ± 1.94	78.98 ± 2.38	85.69 ± 2.21	88.87 ± 2.16
UCTransNet	87.75 ± 1.72	79.93 ± 2.16	86.84 ± 1.96	88.73 ± 2.05
VM-UNet	88.07 ± 1.63	80.11 ± 2.08	87.22 ± 1.87	89.03 ± 1.94
nnWNet	88.51 ± 1.52	80.97 ± 1.96	88.27 ± 1.78	89.16 ± 1.86
HT-Net (ours)	**89** **.37 ± 1.18**	**82.05 ± 1.67**	**89.54 ± 1.73**	**89.40 ± 1.50**

Note: Bold values indicate the best performance among all compared methods.

**Table 5:** Comparison of segmentation performance between HT-Net and baseline methods on the Glas dataset.

Model	mDice	mIoU	mRecall	mPrecision
U-Net	92.19 ± 1.12	85.52 ± 1.54	92.22 ± 1.26	92.21 ± 0.98
UNet++	91.66 ± 1.35	84.59 ± 1.61	91.60 ± 1.48	91.73 ± 1.17
UCTransNet	92.03 ± 1.08	85.26 ± 1.56	92.06 ± 1.19	92.18 ± 1.02
VM-UNet	92.51 ± 0.96	86.07 ± 1.38	92.43 ± 1.04	92.84 ± 0.87
nnWNet	92.74 ± 0.89	86.48 ± 1.17	92.77 ± 0.95	92.73 ± 0.82
HT-Net (ours)	**93.04 ± 0.75**	**86.95 ± 0.99**	**93.03 ± 0.81**	**93.09 ± 0.64**

Note: Bold values indicate the best performance among all compared methods.

**Table 6:** Ablation Study Results on the Camelyon16 Dataset.

Model	mDice	mIoU	mRecall	mPrecision
U-Net	87.09 ± 1.86	79.28 ± 2.31	85.35 ± 2.14	89.44 ± 2.03
Dual-SE	87.93 ± 1.72	80.17 ± 2.17	87.19 ± 1.96	88.62 ± 1.91
Dual-SE-HMSA	88.76 ± 1.43	81.53 ± 1.89	88.31 ± 1.82	89.28 ± 1.69
HT-Net (ours)	**89.37 ± 1.18**	**82.05 ± 1.67**	**89.54 ± 1.73**	**89.40 ± 1.50**

Note: Bold values indicate the best performance among all compared methods.

**Table 7:** Ablation Study Results on the Glas Dataset.

Model	mDice	mIoU	mRecall	mPrecision
U-Net	92.19 ± 1.12	85.52 ± 1.54	92.22 ± 1.26	92.21 ± 0.98
Dual-SE	92.30 ± 1.05	85.70 ± 1.76	92.50 ± 1.13	92.26 ± 0.94
Dual-SE-HMSA	92.61 ± 0.92	86.24 ± 1.61	92.70 ± 0.98	92.55 ± 0.86
HT-Net (ours)	**93.04 ± 0.75**	**86.95 ± 0.99**	**93.03 ± 0.81**	**93.09 ± 0.64**

Note: Bold values indicate the best performance among all compared methods.

## Data Availability

The datasets analyzed in this study are publicly available. The Camelyon16 dataset can be accessed at https://camelyon16.grand-challenge.org/ (accessed on 11 October 2026), and the GlaS dataset at https://warwick.ac.uk/fac/sci/dcs/research/tia/glascontest/ (accessed on 11 October 2026; or via the original publication: [38]). No new data were created in this study. The source code of HT-Net is not publicly available at this time but is available from the corresponding author upon reasonable request.

## Abbreviations

The following abbreviations are used in this manuscript:

WSI	Whole-Slide Image
SLN	Sentinel Lymph Node
MIL	Multiple Instance Learning
SE	Squeeze-and-Excitation
HMSA	Hybrid Multi-Scale Attention
HT-Net	Head–Tail Network
CBAM	Convolutional Block Attention Module
mDice	mean Dice coefficient
mIoU	mean Intersection over Union
